# Thymostimulin versus placebo for palliative treatment of locally advanced or metastasised hepatocellular carcinoma: a phase III clinical trial

**DOI:** 10.1186/1471-2407-10-457

**Published:** 2010-08-24

**Authors:** Matthias M Dollinger, Christine Lautenschlaeger, Joachim Lesske, Andrea Tannapfel, Anna-Dorothea Wagner, Konrad Schoppmeyer, Oliver Nehls, Martin-Walter Welker, Reiner Wiest, Wolfgang E Fleig

**Affiliations:** 1 Department of Medicine (I), Martin-Luther-University Halle-Wittenberg, Germany; 2 Institute of Medical Epidemiology, Biometry and Informatics, Martin-Luther-University Halle-Wittenberg, Germany; 3 Institute of Pathology, Ruhr University Bochum, Germany; 4 Department of Medicine (II), University Hospital Leipzig, Germany; 5 Department of Medicine (I), University Hospital Tübingen, Germany; 6 Department of Medicine (II), University Hospital Frankfurt, Germany; 7 Department of Medicine (I), University Hospital Regensburg, Germany; 8 University of Leipzig Hospitals and Clinics, Leipzig, Germany

## Abstract

**Background:**

Thymostimulin is a thymic peptide fraction with immune-mediated cytotoxicity against hepatocellular carcinoma (HCC) *in vitro *and palliative efficacy in advanced HCC in two independent phase II trials. The aim of this study was to assess the efficacy of thymostimulin in a phase III trial.

**Methods:**

The study was designed as a prospective randomised, placebo-controlled, double-blind, multicenter clinical phase III trial. Between 10/2002 and 03/2005, 135 patients with locally advanced or metastasised HCC (Karnofsky ≥60%/Child-Pugh ≤ 12) were randomised to receive thymostimulin 75 mg s.c. 5×/week or placebo stratified according to liver function. Primary endpoint was twelve-month survival, secondary endpoints overall survival (OS), time to progression (TTP), tumor response, safety and quality of life. A subgroup analysis according to liver function, KPS and tumor stage (Okuda, CLIP and BCLC) formed part of the protocol.

**Results:**

Twelve-month survival was 28% [95%CI 17-41; treatment] and 32% [95%CI 19-44; control] with no significant differences in median OS (5.0 [95% CI 3.7-6.3] vs. 5.2 [95% CI 3.5-6.9] months; p = 0.87, HR = 1.04 [95% CI 0.7-1.6]) or TTP (5.3 [95%CI 2.0-8.6] vs. 2.9 [95%CI 2.6-3.1] months; p = 0.60, HR = 1.13 [95% CI 0.7-1.8]). Adjustment for liver function, Karnofsky status or tumor stage did not affect results. While quality of life was similar in both groups, fewer patients on thymostimulin suffered from accumulating ascites and renal failure.

**Conclusions:**

In our phase III trial, we found no evidence of any benefit to thymostimulin in the treatment of advanced HCC and there is therefore no justification for its use as single-agent treatment. The effect of thymostimulin on hepato-renal function requires further confirmation.

**Trial Registration:**

*Current Controlled Trials ISRCTN64487365*.

## Background

Hepatocellular carcinoma (HCC) currently ranks fifth among the most common malignancies world-wide with a rising incidence in first-world countries [1]. Although treatment options have become more diverse in recent years, improvements in survival rates lack far behind those achieved in other tumor entities [2]. Benefiting most from newer modalities are patients amenable to local therapy, i.e. with intermediate stages of the disease, small tumors and good liver function [3]. In contrast, patients with large tumors, metastases or deteriorating liver function remain without proven standard treatment resulting in a life expectancy of less than 10% at 3 years [5,6]. Most systemic approaches have yielded disappointing results despite major side-effects. Only sorafenib, a new protein kinase inhibitor, has now been shown for the first time to improve survival in patients with Child A cirrhosis [6], leading to new recommendations on design and patient selection in HCC trials [7].

Immunomodulation is another promising strategy against HCC [8]. Thymostimulin - a standardized low-molecular protein fraction containing thymosin-α_1 _and thymic humoral factor [8] - has been demonstrated to induce a selective, dose-dependent, cytotoxic immune reaction against HCC cell lines *in vitro *[9,10]. Based on the experimental data, two single-center phase II trials using thymostimulin in patients with locally advanced and metastasised HCC not amenable to or failing surgical and/or local therapy have been published including one by our group [11,12]. With 63% and 79%, respectively, both depicted excellent tumor control rates even in metastatic disease and virtually no side-effects. The two trials, however, lacked control groups. Thus, we conducted a multi-center, randomised, placebo-controlled phase III study in HCC patients according to the same eligibility criteria at the time. Liver function (Child classification) was used for stratification and subgroup analysis. The aim was to evaluate if the tumor control by thymostimulin observed in the phase II trials would translate into improved survival as compared with best supportive care and placebo.

## Methods

### Eligibility criteria

Patients with histologically proven, locally advanced or metastatic HCC not amenable to or failing established treatment were enrolled; locally advanced tumor was defined as one nodule larger than 5 cm or more than 3 nodules larger than 3 cm in diameter. Pretreatment of the HCC was allowed if tumors had progressed during therapy. However, a treatment-free interval of at least 3 months was required prior to enrollment. Patients between 18 and 80 years of age and a Karnofsky performance status ≥ 60% were eligible. Exclusion criteria were pregnancy/lactation, active second malignancy, severe concomitant disease (e.g. cardiac failure NYHA III-IV, serum creatinine level > 300 μmol/l) or severely decompensated liver function (bilirubin > 5 mg/dl, Child-Pugh > 12). None of the patients received antiviral treatment with interferon during the study period. Ethical approval was obtained from all ethical review boards of the participating centers before study initiation and written informed consent from each patient before entering the study. The study was conducted in accordance with the ethical principles stated in the Declaration of Helsinki and the guidelines on good clinical practice.

### Study design

**Figure 1:**
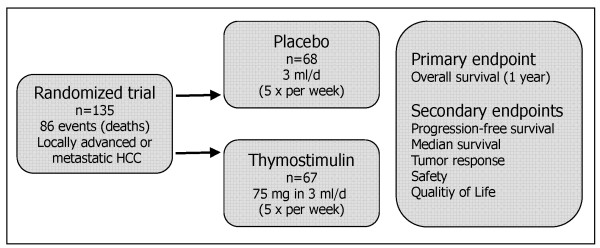
**Schematic presentation of the study design**.

The study was designed as a prospective, randomised, controlled, double-blind, multi-centre phase III trial comparing best supportive care plus thymostimulin with best supportive care plus placebo (trial registration: Current Controlled Trials ISRCTN64487365). Patients were recruited at 12 centres in Germany (see Acknowledgements); all biopsies were centrally reviewed by an experienced pathologist (AT). Primary endpoint of the study was 12-month survival, secondary endpoints overall survival, time to progression and tumor response according to standard Response Evaluation Criteria In Solid Tumors (RECIST criteria) [13], toxicity according to ECOG criteria [14] and quality of life assessed by means of the FACT-Hep questionnaire [15] (Figure 1). A sample size of 110 patients was calculated to detect a 20% improvement in one-year-survival in a two-sided log-rank-test with a significance level of 5% and a power of 90%. Allowing for a drop-out rate of at least 20%, 135 patients were planned to be recruited within 24 months. After inclusion of the last patient, a follow-up period of 12 months was scheduled.

### Therapeutic procedures

Thymostimulin is a licensed immunomodulating drug prepared from an extract of peptides from bovine thymus glands (Thymophysin CytoChemia^® ^25/50). Following removal of high-molecular cell components and proteins, the low-molecular active thymus peptides are isolated and standardized to a defined protein fraction. Before central randomization via fax patients were stratified according to liver function (Child classification) and treatment before study entry (received or not received). They were assigned covariate-adapted to each center to either thymostimulin 75 mg or placebo subcutaneously 5 days a week for one year in addition to best supportive care. Study medication (verum and placebo) was coded and labeled to guarantee blinding with the first five injections performed by a medical doctor. Further injections were administrated by the patients themselves or home care workers and registered in a treatment diary. Drug accountability procedures, reporting of (serious) adverse events and source data verification were implemented as part of the trial monitoring according to standard operating procedures conducted by external monitors. Treatment with the study medication was continued for one year or until one of the following criteria was met: disease progression, decompensation of liver function (bilirubin > 5 mg/dl, INR ≥ 2.3) or performance status (Karnofsky score < 50%), unacceptable toxicity, patient refusal or incompliance.

### Pre-treatment and follow-up evaluation

Pre-treatment and follow-up evaluation included a complete medical history, physical examination, blood count and chemistry as well as performance status. Risk factors and severity of liver disease according to Child-Pugh status as well as prior treatment modalities were recorded at baseline. Tumors were assessed by abdominal ultrasound, chest X-ray and either dynamic computerized tomography (CT) or magnetic resonance imaging (MRI). Other imaging techniques such as radionuclide scans were deployed as clinically indicated. Okuda, CLIP and BCLC classifications were used for staging. Follow-up investigations without imaging were conducted every 6 weeks including survival data as well as documentation of concomitant therapies and toxicity of the medication. Tumor response using CT or MRI scanning was evaluated every 3 months according to RECIST criteria [13]: complete response = disappearance of all demonstrable disease; partial response = decrease ≥ 50% of the longest diameter of the target lesion without worsening of all other disease; stable disease = no significant change in disease [decrease < 50% or increase < 25% without new lesions]; progressive disease = increase ≥ 25% of the longest diameter of the target lesion or new lesions). In addition, quality of life assessed by means of the FACT-Hep questionnaire [15] was also measured every 3 months. Follow-up was scheduled for at least one year after enrollment, followed by three-monthly assessments in surviving patients.

### Statistical analysis

All analyses were based on the intention-to-treat (ITT) patient population. Baseline characteristics were expressed as median [range or 95% CI] for continuous variables and percentages (of all patients randomised) for categorical variables. Survival time and time to progression were calculated from the time of randomisation to the date of death or date of progression, respectively. Univariate survival curves for baseline predictors were established with the Kaplan-Meier method and quantitatively expressed as median survival time [95% CI]. As predefined in the study protocol, a stepwise backward Cox's regression analysis of survival was used to adjust the treatment effect according to relative risks imposed by baseline predictors. The following variables were chosen for the first model: age, gender and Karnofsky performance status; the presence of liver cirrhosis and Child classification; Okuda, CLIP and BCLC classification; AFP level, multifocal tumor manifestation, ascites, vascular invasion and extrahepatic metastases; treatment with thymostimulin or placebo and treatment with other therapeutic modalities before study entry. The frequency of adverse events was compared between groups using Chi-square tests. All calculations were done with the SPSS package (version 15.0).

## Results

### Patients

A total of 135 patients were enrolled between October 2002 and March 2005, and randomly assigned to treatment with thymostimulin (n = 67) or placebo (n = 68). Detailed demographic data and tumor-related characteristics are depicted in Table 1. Most patients had liver cirrhosis, and the majority of tumors was staged as advanced HCC (Okuda stage II; CLIP 2-3 points; BCLC stage C) with multinodular tumor growth, vascular invasion and extrahepatic spread. Less than a third of patients had been treated prior to enrollment with surgical resection (R1 or R2 resection), percutaneous ethanol injection (PEI; range 1-5 sessions), transarterial chemoembolisation (TACE; range 1-7 sessions) or systemic hormone- and/or chemotherapy (somatostatin in 7/14 cases; single or combination therapy with tamoxifen, doxorubicin or platin derivates), but suffered from tumor progression. All baseline characteristics were well balanced between the two groups.

**Table 1:** Baseline characteristics of patients

Baseline characteristics	Placebo n = 68	Thymostimulin n = 67
***Patients***		
Male/female *(%)*	82/18	84/16
Median age *(years (range))*	63 (39-76)	63 (48-79)
Median body weight *(kg (range))*	80 (39-108)	79 (58-112)
Mean Karnofsky score *(% (range))*	87 (60-100)	89 (60-100)

***Cause of liver disease (%)***		
Alcohol abuse	47	55
HBV/HCV	21	18
Other	32	27

***Stage of liver disease (%)***		
Liver cirrhosis	90	85
Child classification: A or no cirrhosis/B/C	63/28/9	65/32/3

***Tumor stage (%)***		
Okuda stage I/II/III	32/56/12	35/54/11
CLIP score 0/1/2/3/4-6	0/18/35/25/22	0/15/29/29/27
BCLC stage A/B/C/D	0/16/69/15	0/22/66/12

***α-FP (%)***		
< 400 ng/ml	57	57
> 400 ng/ml	43	43

***Tumor characteristics (%)***		
Ascites	46	37
Portal vein thrombosis	32	42
Multifocal tumor manifestation	88	91
Extrahepatic metastases	40	45

***Hepato-renal laboratory parameters***		
Mean urea *(mg/dl [95% CI])*	4.3 [2.8-5.8]	3.7 [2.8-4.5]
Mean creatinine *(mg/dl [95% CI])*	0.4 [0.3-0.6]	0.5 [0.4-0.6]
Mean sodium *(mmol/l [95% CI])*	137 [136-138]	137 [136-138]
Mean albumin *(g/l [95% CI])*	27 [23-31]	29 [26-32]

***Previous treatment (%)*** *(combination possible)*		
none	68	72
Surgery	15	9
Percutaneous ethanol injection (PEI)	4	2
Transarterial chemoembolisation (TACE)	25	20
Systemic hormone- and/or chemotherapy	12	9

**Figure 2:**
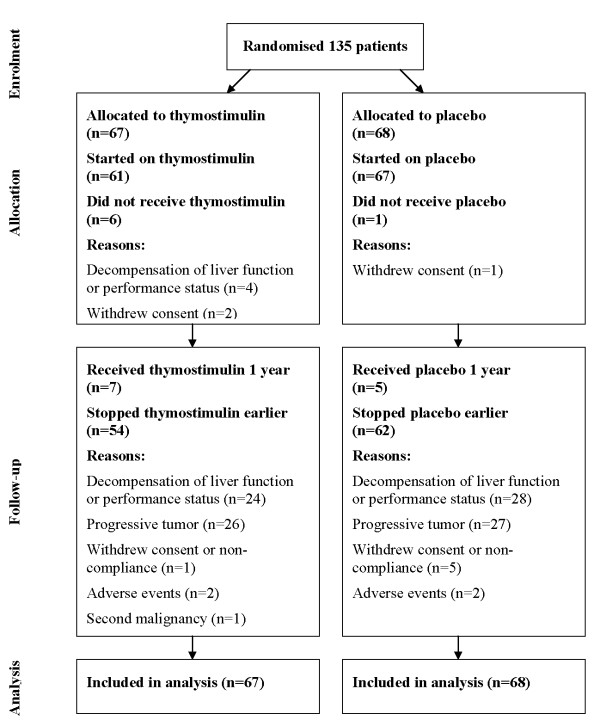
**Flow diagram of the phase III trial according to CONSORT guidelines**.

Following randomisation, 6 patients in the thymostimulin group and one patient in the placebo group did not receive the allocated treatment (Figure 2). Their data were included in the intention-to-treat analysis; a subgroup analysis excluding these patients did not reveal a different outcome (data not shown). The remaining patients received treatment as scheduled, in most cases until disease progression or deterioration of liver function or performance status. One patient in each group received salvage therapy after tumor progression, in the placebo group TACE, in the thymostimulin group tamoxifen. Secondary tumors requiring alternative therapy were also diagnosed one in each group; in the thymostimulin group, treatment of the patient had to be stopped early due to a hypopharyngeal cancer, in the placebo group the secondary urothelial cancer was detected after 12 months of treatment of the patient. Only 12 patients (thymostimulin, n = 7 [10%]; placebo, n = 5 [7%]) received the study medication for a whole year. At the time of analysis, all patients had stopped their allocated treatment. The median follow-up was 5.2 months [95% CI 4.0-7.3] in the thymostimulin group and 5.6 months [95% CI 3.6-7.3] in the placebo group, the median length of treatment 3.3 months [range 0 to 12 months] for both groups.

### Survival and tumor response

**Figure 3:**
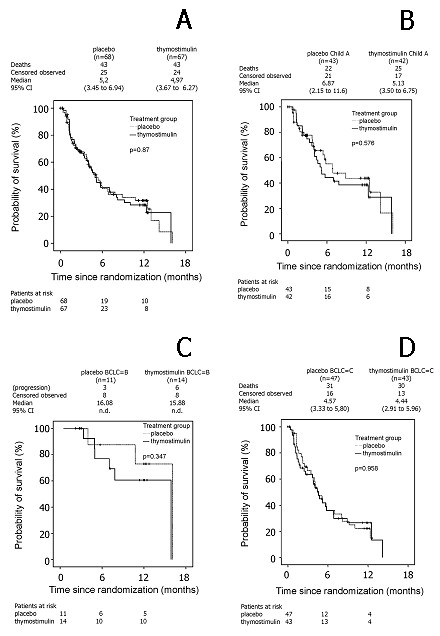
**Estimate of overall survival**. Kaplan-Meier graph showing probability of overall survival over time in percentage of patients randomised (- survival function, + censored): A) all patients; B) subgroup analysis Child A patients; C) subgroup analysis BCLC B patients; D) subgroup analysis BCLC C patients.

For the thymostimulin group, the univariate probability of survival at 6 and 12 months was 42% [95% CI 29-55] and 28% [95% CI 17-41], respectively, with a median survival time of 5.0 months [95% CI 3.7-6.3]. There was no significant difference in univariate survival compared with the placebo group with a 6- and 12-month survival of 40% [95% CI 27-54] and 32% [95% CI 19-44], respectively, and a median survival time of 5.2 months [95% CI 3.5-6.9; p = 0.87; HR = 1.04 [95% CI 0.7-1.6]]. The Kaplan-Meier curve for overall survival is shown in Figure 3A.

**Figure 4:**
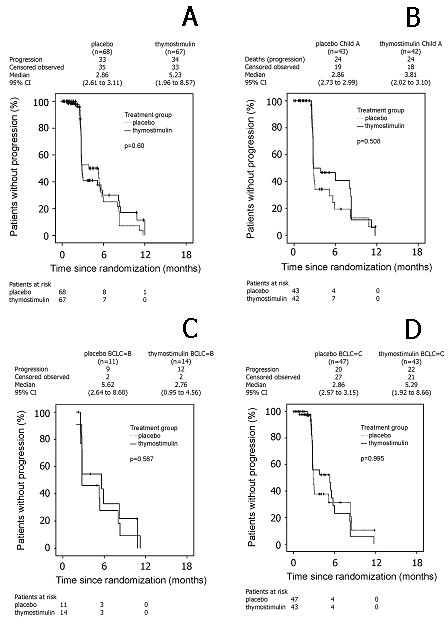
**Estimate of time to progression**. Kaplan-Meier graph showing time to progression in percentage of patients randomised (- survival function, + censored): A) all patients; B) subgroup analysis Child A patients; C) subgroup analysis BCLC B patients; D) subgroup analysis BCLC C patients.

The analysis of the objective tumor response showed no complete response in any of the patients. 22 [33%] out of 67 patients in the thymostimulin group achieved stable disease or a partial response (disease-control rate) in the first 6 months followed by progressive disease thereafter. There was, however, a similar tumor response rate in the placebo group in 20 [29%] out of 68 patients, and time to progression did not statistically differ between groups [p = 0.60, HR = 1.13 [95% CI 0.7-1.8]] with a median of 5.3 months [95% CI 2.0-8.6; thymostimulin] and 2.9 months [95% CI 2.6-3.1; placebo], respectively (Figure 4A).

As predefined in the study protocol, an exploratory Cox's regression analysis of survival and time to progression was used to adjust the treatment effect of thymostimulin or placebo according to the relative risks imposed by baseline characteristics. Thus, adjustment for age, gender, Karnofsky performance status, presence of liver cirrhosis and stage of liver disease (Child classification), tumor stage (Okuda, CLIP and BCLC classification), invasive tumor phenotype (vascular invasion or metastases) or treatment prior to enrollment did not reveal a therapeutic effect of thymostimulin in any subgroup of patients. Kaplan-Meier curves for overall survival and time to progression in patients with Child A cirrhosis or tumor stage BCLC B and C are shown in Figure 3B-D and Figure 4B-D, respectively.

### Safety

Compliance with the allocated treatment was high with an average of 94% and 92% of patients receiving all 5 injections a week in every 6 week-cycle in the thymostimulin and placebo group, respectively (*n.s*.). While skin reactions following injection are the only known side-effects of thymostimulin [11,12], it has been shown to reduce chemotherapy-induced toxicity, in particular myelosuppression and infectious episodes [16]. In our study, 219 adverse events were reported - 95 in the placebo and 124 in the thymostimulin group (*n.s*.). Skin reactions caused the early withdrawal from the trial of two patients on thymostimulin and one patient on placebo (*n.s*., Table 2). One more patient on placebo withdrew due to adverse events, i.e. diarrhea after injection (Figure 2). 139/219 adverse events were classified as serious - 85 in the placebo and 54 in the thymostimulin group. Using Chi-square-tests, we found a lower incidence of renal failure, ascites and dyspnoea in patients treated with thymostimulin (Table 2). In contrast, hematological or infectious complications were equally common in both groups.

**Table 2:** Comparison between groups of (serious) adverse events (AE) graded according to ECOG criteria and occurring in more than 2% of all patients (n = 135; significance level p < 0.05)

ECOG toxicity criteria	Placebo (n = 68)				Thymostimulin (n = 67)				p-value
	Grade 1	Grade 2	Grade 3	Grade 4	Grade 1	Grade 2	Grade 3	Grade 4	single tests
***Haematology***									
Anaemia		1		1	2				*0.9880*

***Constitutional symptoms***									
Fatigue		2	2	1		1	1		*0.2525*

***Dermatology***									
Injection site reaction		1			1	3			*0.1663*

***Pain***									
Abdominal pain	1			2	1			2	*0.9852*
Arthralgia			1		3				*0.3029*

***Infection***									
Infection without neutropenia	3		4	1	1	3	3		*0.8077*

***Gastrointestinal/Hepatic***									
Diarrhoea	1		2		2	2			*0.6831*
Hyperbilirubinaemia				2				1	*0.5681*
Elevated transaminases		1	2	1	2	1	2		*0.7128*
GI haemorrhage			4	2			4	6	*0.2728*
Ascites (without renal failure)		1	14	3		2	6		**0.0323**

***Renal***									
Elevated creatinine or urea	5	2			1			1	*0.0887*
Renal failure (with ascites)			7	5				2	**0.0052**

***Pulmonary***									
Dyspnoea		3	4			1			**0.0303**
Pleural effusion			6				1		*0.0548*

***Neurology***									
Encephalopathy				6				5	*0.7726*

### Quality of life

Quality of life was assessed by means of the FACT-Hep questionnaire at baseline and every 6 weeks thereafter. There was no difference between groups in any of the questionnaire's subdomains at any time point.

## Discussion

This is the first randomised controlled trial analysing the effect of thymostimulin or similar thymic extracts containing thymosin-α_1 _on HCC growth and survival of patients. We found no difference in either 12-month/overall survival or time to progression between the treatment and placebo group when using the compound as a single-agent regimen. Despite its good safety profile, thymostimulin failed to prevent the occurrence of infectious complications as previously reported, although the incidence of renal decompensation and ascites was reduced as compared with placebo.

There has been a renewed interest in compounds containing thymosin-α_1 _over the past years for their potential immune-stimulating and anti-tumorous properties resulting in ongoing large phase II/III trials in melanoma or hepatitis C patients [17-19]. The underlying mechanism of action of these compounds is thought to be an increase of proinflammatory cytokines, T cell proliferation and differentiation as well as antigen expression on tumor cells resulting in the induction of a tumor-specific cytotoxicity [19,20]. Indeed, preliminary phase II trials of thymostimulin in patients with HCC were notable for their tumor response rate and the occurrence of complete responses, resulting in a median overall survival of up to 11.5 months [11,12]. This observation could not be repeated in the current phase III trial. Rather, thymostimulin had no beneficial effect on tumor progression as compared with placebo, and thus did not improve survival.

Although superior in design than the phase II trials, there are several limitations to our study originating in the concept from 2002. Based on the previous experiences, patients now generally classified as at the intermediate, advanced and terminal stage of their disease (BCLC classification) were enrolled resulting in a median survival of only 5 months and early withdrawal due to decompensation of liver function or performance status in more than a third of patients. In his recent recommendations, Llovet et al. clearly discouraged study designs including patients at different stages of the disease, in particular regarding liver function [7]. Survival in Child B and C cirrhosis may be too short to capture any benefit from the trial medication. As intended in our study protocol, we stratified patients prior to randomisation according to liver function (Child classification) and previous treatment for HCC (received or not received). Results in the final analysis were additionally adjusted for baseline characteristics including liver function, tumor stage and tumor phenotype such as vascular invasion or metastases. We found no effect of thymostimulin in any of the subgroups, but overall survival was better in Child A patients, although not as good as in the SHARP trial with its more stringent inclusion criteria [6]. Thus, the limitation in design has to be acknowledged for the interpretation of our results, and should be obsolete in future trials.

In view of the advanced stage of disease of many of the patients, intervals between assessments of tumor response by CT or MRI scanning every three months may have been chosen too long, too. Differences in tumor progression between the two groups within the first 3 months, in particular, might have not registered confounding the secondary endpoint time to progression. Finally, a majority of patients featured alcoholic cirrhosis as underlying liver disease, only a minority viral hepatitis. Although uncommon for many other regions in the world and therefore potentially limiting comparability, the distribution reflects the origin of the patients in Northern Europe and matches official observations of the German Federal Statistical Office [21]. Despite the limitations of the study design, however, we found no convincing evidence for a therapeutic effect of thymostimulin used as a single agent, in particular with regards to the most stringent study endpoint overall survival. A potential impact as "biological response modifier" [17] as part of a combination therapy, recently suggested in two promising phase II trials using thymosin-α_1 _containing compounds in combination with TACE for advanced HCC [22,23], will have to be confirmed in a controlled trial.

In addition to their anti-tumorous potential, thymosin-α_1 _containing compounds have also been associated with improved immune function and a reduction of infectious complications [24]. Only skin reactions following injection are known side-effects [18,19], while several studies demonstrated their ability to prevent myelosuppression and infections during chemotherapy [16,25]. In our study, most adverse events could be related to the progressive tumor and subsequently deteriorating liver function of the patients. In this cohort, thymostimulin failed to reduce infections, bearing in mind that neutropenia did not occur as a complication of the disease. Interesting, but remaining a mere observation in a study not designed to study this endpoint, is the lower incidence of renal failure and ascites in patients treated with thymostimulin as compared with placebo. A similar effect has only been shown once for thymosin fraction 5 (TF5) in an animal model of experimentally induced uremia [26].

## Conclusions

In conclusion, our placebo-controlled, double-blind and randomised study found no evidence of a survival benefit or lasting tumor response in patients with locally advanced or metastasised HCC treated with thymostimulin. Thus, it cannot be recommended as single-agent treatment. This does not preclude testing thymostimulin or similar compounds as part of a combination therapy, although better understanding of their potential mode of action is certainly needed in doing so.

## Abbreviations

AFP: α-fetoprotein; CI: Confidence interval; CT: computerized tomography; ECOG: Eastern Cooperative Oncology Group; MRI: magnetic resonance imaging, n.s.: not significant; HCC: Hepatocellular carcinoma; RFTA: radiofrequency thermal ablation; PEI: percutaneous ethanol installation; TACE: transarterial chemoembolisation; WHO: World Health Organization

## Competing interests

This study was supported by Cytochemia AG (Ihringen, Germany), who provided study medication and funding for project and data management. Final decisions for all aspects of the study rested with the principal investigator (WEF). Data analysis and patient recruitment were performed independently of the funding source. The authors were not paid for writing the manuscript or any other activity regarding this trial and have no financial interest in Cytochemia AG.

## Authors' contributions

MMD analyzed the data, drafted and finalized the manuscript, and coordinated its submission. CL performed the statistical analysis. LJ helped drafting the clinical protocol and enrolled patients. AT did the central pathological review. ADW helped drafting the clinical protocol and revised the report. KS, ON, MW and RW revised the report and enrolled patients. WEF conceived the study and was the principle investigator. All authors read and approved the final manuscript.

## Pre-publication history

The pre-publication history for this paper can be accessed here:

http://www.biomedcentral.com/1471-2407/10/457/prepub
